# Application of four-section approach for prenatal diagnosis of Pierre robin sequence

**DOI:** 10.1007/s10396-025-01556-x

**Published:** 2025-08-22

**Authors:** Xiuling Li, Lingyan Liu, Fang Yan, Xiang Yang, Guanghui Xiu, Xudong Dong

**Affiliations:** 1 Faculty of Life Science and Technology, Kunming University of Science and Technology, Kunming, 650500 Yunnan Province People’s Republic of China; 2 Department of Obstetrics, The First People’s Hospital of Yunnan Province, The Affiliated Hospital of Kunming University of Science and Technology, 157 Jinbi Road, Xishan District, Kunming, 650032 Yunnan Province People’s Republic of China; 3 Medical School, Kunming University of Science and Technology, Kunming, 650500 Yunnan Province People’s Republic of China; 4 Department of Intensive Care Unit, The Affiliated Hospital of Yunnan University (The Second People’s Hospital of Yunnan Province), No. 176 Qingnian Road, Wuhua District, Kunming, 650021 Yunnan Province People’s Republic of China

**Keywords:** Pierre robin sequence, Prenatal diagnosis, Micrognathia, Cleft palate, Glossoptosis

## Abstract

**Purpose:**

This study aimed to evaluate the effectiveness of the four-section approach using two-dimensional sonography in diagnosing Pierre Robin sequence (PRS) during second-trimester screening.

**Methods:**

A prospective study was conducted on low-risk pregnant women undergoing routine mid-trimester screening. Cases with suspected micrognathia prenatally were included and examined using the four-section approach. Initially, we measured the inferior facial angle (IFA) for fetuses suspected of having micrognathia. Subsequently, in the oblique coronal section via the oral fissure, we examined the continuity of the hard and soft palate line. Finally, dynamic scanning of both the sagittal and coronal sections of the mandible was performed to confirm whether the echogenic tongue was displaced posteriorly. All fetuses diagnosed with PRS were followed up through autopsy and postnatal evaluation.

**Results:**

Forty-three fetuses were initially subjectively suspected of having micrognathia by sonographers. After objective IFA measurement, 25 cases had an IFA < 50°, while 18 had an IFA > 50°. Among the 25 cases with IFA < 50°, we identified 13 cases of PRS with micrognathia, cleft palate (CP), and glossoptosis. However, one case failed to be diagnosed prenatally because it had micrognathia and CP but no glossoptosis. Eleven cases had neither CP nor glossoptosis yet exhibited other malformations. Among the 18 fetuses with IFA > 50°, 15 cases were normal, while three cases had other deformities. In this study cohort, no false-positive results were found. The four-section approach for diagnosing PRS showed a sensitivity of 92.9%, a specificity of 100%, a positive predictive value of 100%, and a negative predictive value of 91.7%.

**Conclusion:**

The four-section method proved highly effective in assessing PRS during second-trimester sonographic scans. The combined evaluation of micrognathia and glossoptosis can remarkably enhance the accuracy of prenatal PRS diagnosis.

**Supplementary Information:**

The online version contains supplementary material available at 10.1007/s10396-025-01556-x.

## Introduction

Pierre Robin sequence (PRS) is known as a group of congenital malformations characterized by micrognathia, glossoptosis, and airway obstruction [1, 2], approximately 80% of which are associated with U-shaped cleft palate (CP) [3, 4]. PRS occurs relatively rarely, with an incidence rate ranging from 1/8500 to 1/14 000 of live births [5, 6]. Previous reports indicate that the mortality rate of PRS ranges from 2 to 26% [7, 8]. PRS is usually classified into nonsyndromic (58%) and syndromic (42%) categories, depending on whether it co-occurs with other syndromes [9]. More than 50 syndromes have been described in association with PRS [10]. The three most prevalent syndromes, accounting for 65% of cases, are Stickler syndrome, velocardiofacial syndrome, and Treacher-Collins syndrome [11]. The nonsyndromic group can be further subclassified into isolated PRS and PRS with additional anomalies (associated PRS) [12–14]. Associated PRS coexists with various malformations, particularly those affecting the central nervous system and skeletal system [11]. When PRS is suspected during prenatal diagnosis, it is essential to screen for other concomitant malformations. In fact, the prognosis of children with isolated PRS differs significantly from that of those with syndromic or associated PRS. The latter group has a higher mortality rate and poorer developmental outcomes [15]. Magnetic resonance imaging (MRI) and ultrasound are widely recognized as primary tools for the prenatal diagnosis of PRS. Fetal MRI allows for direct visualization of the fetal tongue’s position within the oral cavity, its relationship with the palate, and precise assessment of tongue retroposition and airway occlusion [16, 17]. However, due to its cost, limited availability, and potential risks to the fetus, MRI is not a routine screening method. It serves as a supplementary option when ultrasound results are inconclusive. With the continuous improvement in the accuracy of ultrasound equipment, prenatal ultrasound has proved highly effective in diagnosing PRS. It plays a crucial role in perinatal clinical management and genetic counseling.

Micrognathia is one of the key features of PRS. Historically, the prenatal diagnosis of fetal micrognathia relied on visual inspection of midsagittal ultrasound images of the fetal facial profile, without the use of a standardized metric, resulting in low sensitivity [18, 19]. To enhance diagnostic accuracy, several objective techniques have been developed and implemented to quantify micrognathia during prenatal examinations. These include measuring the jaw index, mandibular length, fronto-naso-mental angle (FNMA), and inferior facial angle (IFA), among others [19–24]. Among these, an IFA of less than 50° serves as a reliable indicator for micrognathia diagnosis, with 100% sensitivity and 98.9% specificity [23–25]. The prenatal diagnosis of CP remains challenging due to the lack of a standardized approach for evaluating the hard palate. Indirect ultrasound signs of CP include the “equals sign,” “superimposed-line absence” sign, and “posterior reflexed angle” of the hard palate [26–28]. The axial transverse view of the hard palate is considered a practical method for assessing the fetal hard palate via 2D during the second-trimester scan [27]. Numerous prenatal ultrasound specialists include this perspective in their routine fetal assessments. Lately, the detection rate of CP has gone up, reaching approximately 65% in certain centers [29, 30]. Besides micrognathia, glossoptosis is another key diagnostic indicator of PRS. Previous studies have reported subjective assessment methods for glossoptosis [16, 31, 32]. Glossoptosis is characterized as a tongue displaced backward, which cannot reach the anterior mandibular alveolar ridge in the fetal sagittal view. Diagnosing glossoptosis through prenatal ultrasound is quite challenging. It demands dynamic and careful observation and is prone to misdiagnosis. Glossoptosis is also a main factor leading to subsequent airway obstruction and feeding problems in affected kids. As a result, the antenatal diagnosis of fetal glossoptosis is of great clinical significance.

This article comprehensively describes the four-section scanning method for PRS diagnosis and analyzes the ultrasonographic features of PRS cases. The purpose is to enhance the prenatal detection rate of PRS, thereby facilitating safe parturition and improving perinatal management.

## Materials and methods

### Study subjects

A prospective study was performed from January 2017 to December 2022 at the First People’s Hospital of Yunnan Province for pregnant women who underwent routine second-trimester sonographic screening. The inclusion criterion was cases with prenatal suspicion of micrognathia. We conducted an in-depth analysis of the prenatal data, which included ultrasonic characteristics, associated anomalies, chromosomal examination results, as well as perinatal and neonatal outcomes in cases of PRS. All cases included were confirmed by autopsy or postpartum examination.

## Methods

### Ultrasound detection

**Fig. 1:**
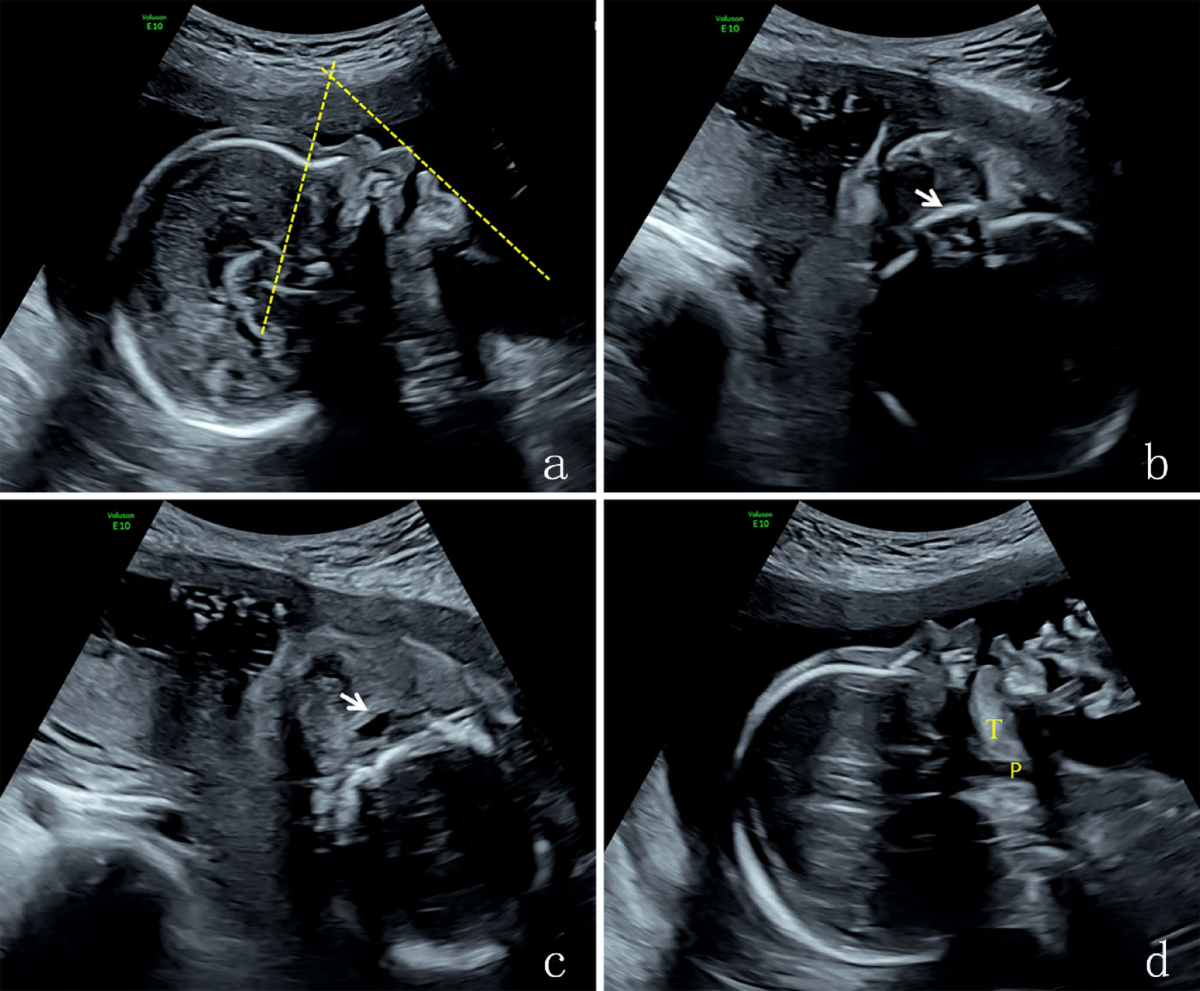
Four sections of a normal fetus: **a** The median sagittal section, mandibular angle was 61°; **b** The oblique coronal section of hard plate via the oral fissure, it shows the strong echo of the hard palate plate, which is continuous and complete (the arrow indicates the hard palate); **c** The obliquely coronal section of soft palate through oral fissure, it shows that a complete soft palate (the arrow indicates the equal sign of soft palate); **d** The sagittal section of mandible, it shows the lingual front reached the inferior alveolar ridge and the pharyngeal space was normal.T: tone, P: pharynx

The sonographic examination was carried out transabdominally using high-resolution ultrasound equipment (Voluson E10 and Voluson E8 with convex probes C1-5-D and C4-8-D, 3.5-5.0 MHz), after which the fetal anatomy was evaluated for malformations according to the guidelines recommended by the International Society of Ultrasound in Obstetrics and Gynecology (ISUOG) [33] **(**Fig. 1**)**.

Suspected micrognathia was defined subjectively by the sonographer when the mandibula appeared to be small or retropositioned [16]. We measured the IFA in the median sagittal section of the face for fetuses with suspicion of mandibular dysmorphology. IFA is an objective method for assessing poor mandibular development, which is measured as the angle between a line vertical to the forehead and a line joining the mentum and protrusive lip. IFA < 50° has been recommended as the diagnostic criterion for micrognathia [24]. Subsequently, we identified disruptions in the hard and soft palate using the oblique coronal section via the oral fissure. Dynamic scanning of the sagittal and coronal sections of the mandible was performed to observe the position of the fetal tongue in the oral cavity. In the sagittal sections of the mandible, we focused on determining whether the front edge of the tongue reached the inferior alveolar ridge, if the root of the tongue extended to the posterior pharyngeal wall, whether the pharyngeal space narrowed, and whether the epiglottis movement was restricted. In the coronal section of the mandible, we checked whether the fetal tongue filled the pharyngeal cavity. The current diagnostic criteria for PRS encompass a combination of micrognathia, glossoptosis, and respiratory obstruction. In some cases, PRS is accompanied by a CP [34].

### Follow-up and review of the images of the PRS cases

Genetic counselling and fetal karyotyping were recommended for all the PRS cases. Some patients opted for pregnancy termination on the basis of the complex malformations, karyotyping, and chromosomal microarray analysis (CMA) results. The appearance and oral cavity of the induced fetuses were further examined. For pregnancies that continued, close monitoring was maintained until birth. Neonatologists then conducted a comprehensive evaluation of the neonates’ appearance and oral cavity. Postpartum or autopsy outcomes were followed up.

We thoroughly summarized and discussed the latest findings on prenatal ultrasonic characteristics, associated anomalies, chromosomal examination results, and perinatal and neonatal outcomes in PRS cases. Additionally, the reasons for failing to diagnose were explored and analyzed.

## Results

### Study population

**Fig. 2:**
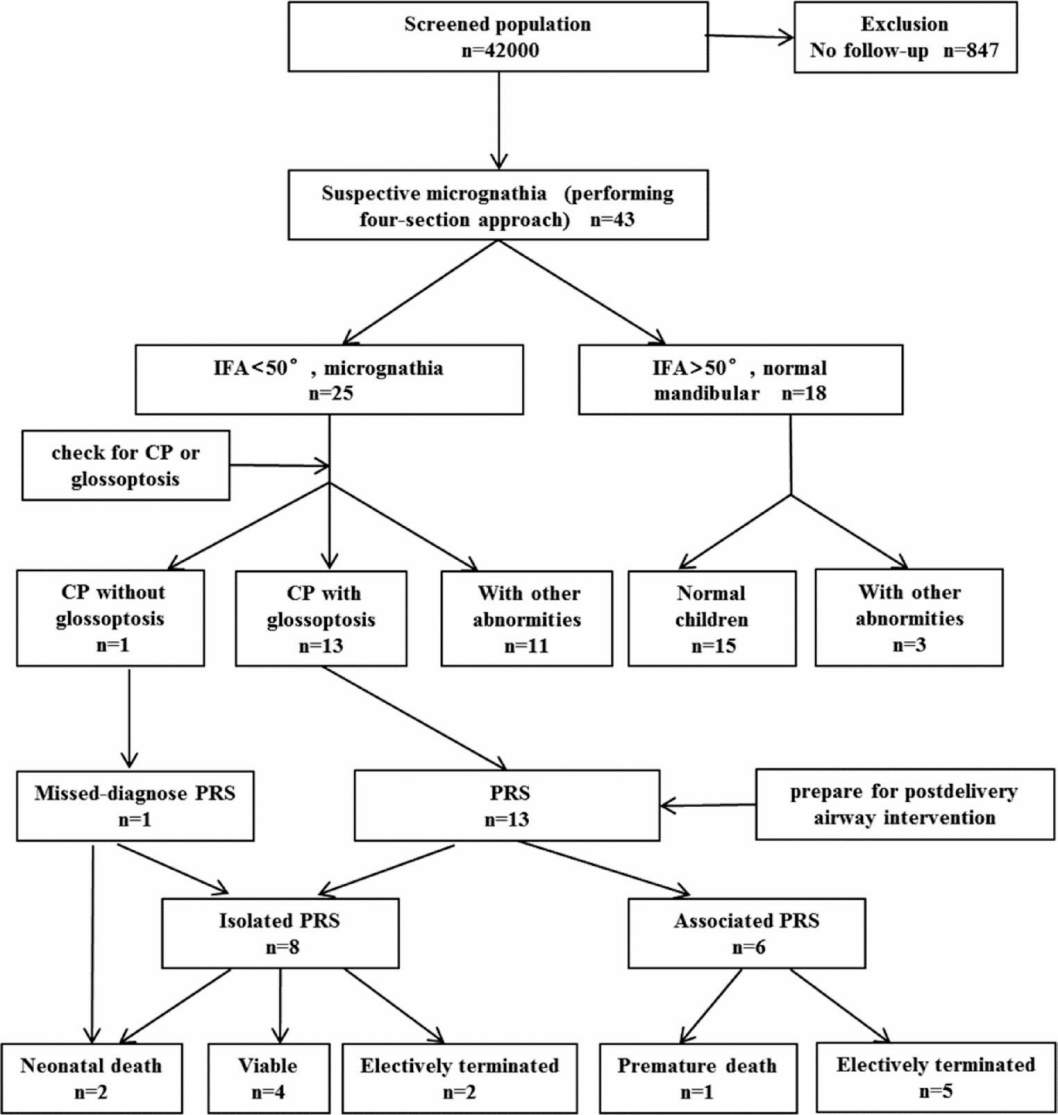
Study population flow chart. CP: cleft palate, IFA: inferior facial angle, PRS: Pierre Robin sequence

In a cohort of 42,000 cases subjected to prenatal ultrasound screening, 847 fetuses were excluded owing to the lack of follow-up outcomes. The median maternal age was determined to be 29.5 years (range 18–51 years). The mean gestational age calculated from ultrasound examination was 24.5 weeks (range 20–28 weeks). Forty-three fetuses were subjectively interpreted as having micrognathia during the initial sonographic examination. After reassessing with the objective measure of the IFA, 25 cases met the objective criteria for micrognathia. The incidence of micrognathia in our cohort was 0.06%. Among the 25 cases with micrognathia, 13 (52.0%) had CP and glossoptosis and were diagnosed with PRS. One case (4.0%) had CP but no glossoptosis, which was exactly the fetus that went undetected prenatally in this study. Eleven cases (44.0%) had neither CP nor glossoptosis but presented other malformations. Regarding the 18 fetuses initially suspected of having micrognathia but not meeting the objective criteria, their IFA values were greater than 50°. Of these, 15 (83.3%) were born normal, while three (16.7%) had other deformities (Fig. 2).

After postnatal or post-mortem confirmation, 13 cases of PRS were identified at prenatal ultrasound, yet one case was undiagnosed prenatally. In this cohort, no false-positive results were detected. The four-section approach for diagnosing PRS demonstrated a sensitivity of 92.9%, a specificity of 100%, a positive predictive value of 100%, and a negative predictive value of 91.7%. The incidence of PRS was 0.03%. Among these cases, eight (57.1%) presented with isolated PRS, while six (42.9%) were found to have associated deformities.

### Ultrasound characteristics of 14 cases of PRS

**Fig. 3:**
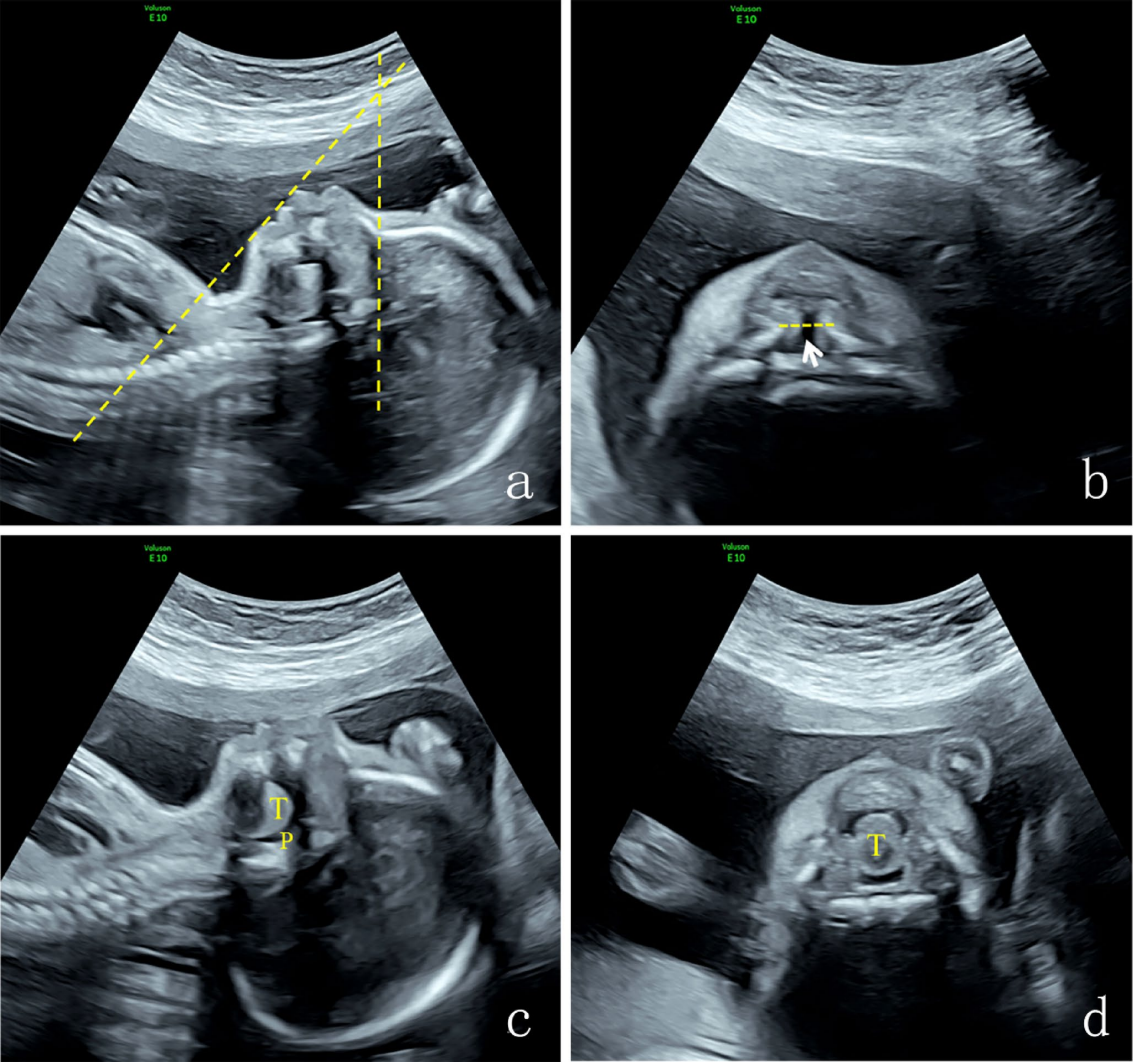
Four sections of a PRS case: **a ** shows micrognathia, with obvious retraction of the mandible, and the IFA was 38° ; **b ** shows interruption of the hard palate (arrow shows cleft palate ); **c ** shows the tongue filling oropharyngeal cavity; **d ** shows that the tongue fell back to the posterior pharyngeal, and the lingual front could not reach the inferior alveolar ridge. T: tone, P: pharynx, IFA: inferior facial angle

**Fig. 4:**
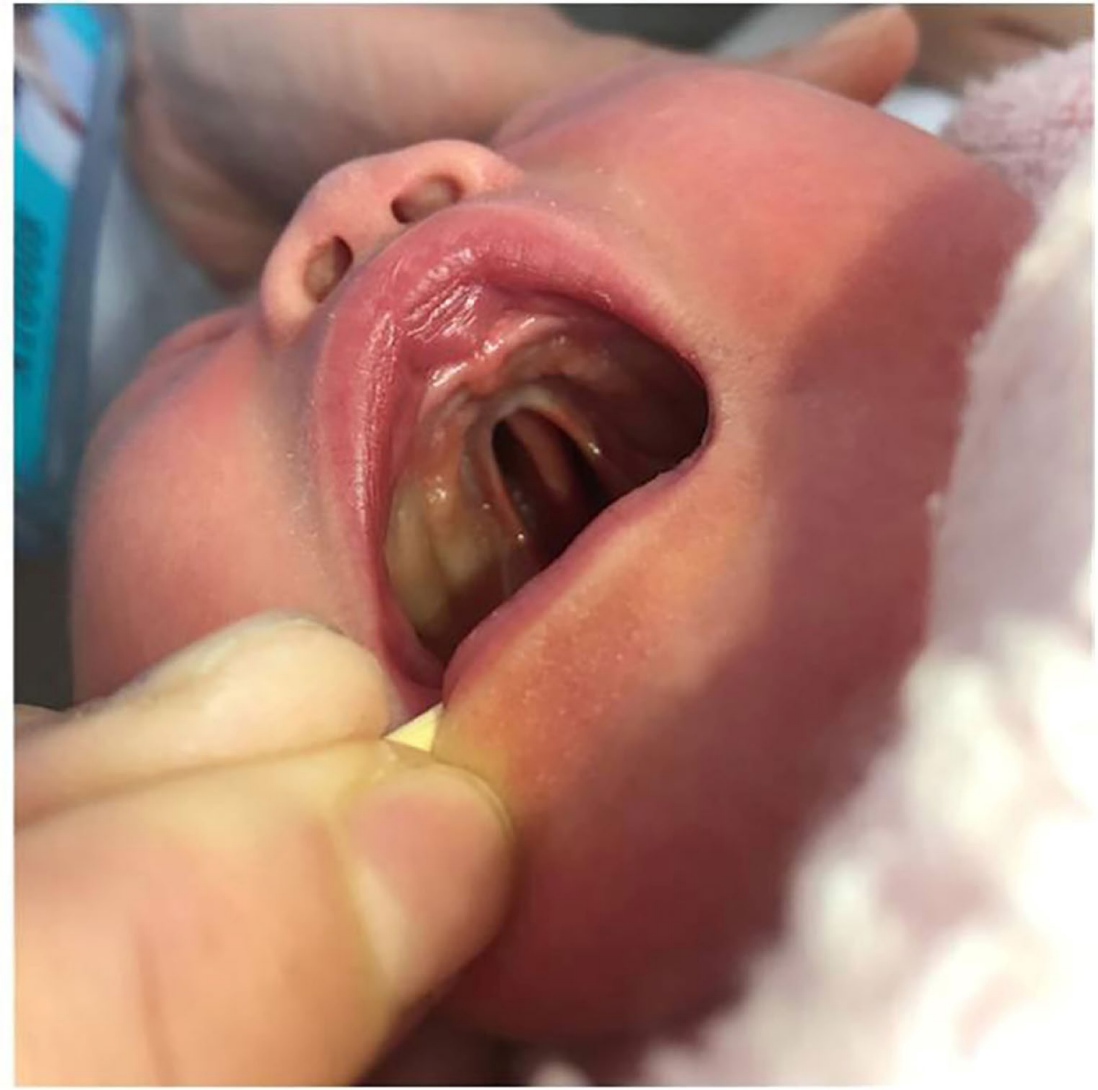
Full-term delivery, U-shaped CP was visible, CP: cleft palate

The 14 cases of PRS exhibited different degrees of micrognathia or mandibular retraction on prenatal ultrasound with an IFA < 50° (Fig. 3a). The average IFA of PRS fetuses with micrognathia was 39.8° (range 26°-48°), as presented in Table 1. All 14 PRS fetuses had incomplete CP without lip or alveolar clefts. Four of these clefts displayed a “U” shape. In the oblique coronal section of the palate, partial disruption was observed in the hard and soft palate (Fig. 3b). Glossoptosis was found in 13 cases prenatally, which was expressed as the tongue tip being distant from the superior alveolar process in the sagittal section of the mandible with the tongue prolapsing into the hypopharyngeal space and forming a narrowed pharyngeal cavity, which restricted the movement of the epiglottis (Fig. 3c, **Video 1**,** Video 2**). The tongue moved backward and downward, nearly filling the oropharyngeal cavity in the oblique coronal section of the mandible. During swallowing, the tongue’s movement within the pharyngeal cavity was not significant (Fig. 3d, **Video 3**). The case with a missed diagnosis was prenatally identified as micrognathia and incomplete CP, but glossoptosis failed to be diagnosed due to the fetal head-neck flexion blocking the visualization of the tongue’s position. It was diagnosed as PRS after the postpartum examination (Fig. 4).

**Table 1:** Prenatal ultrasound characteristics and outcomes of 14 fetuses with PRS

Case number	Maternal age (years)	GW (week)	Polyhydramnios	IFA	Prenatal diagnosis	Outcomes of postpartum exam or autopsy	Associated deformities	Chromosomal findings	Clinical outcomes
1	24	23 + 1	+	44°	PRS	Micrognathia, U-shaped CP, soft cleft, glossoptosis	–	Normal NIPT, karyotype, and CMA	Full-term delivery, viable
2	35	23 + 6	−	39°	PRS	Micrognathia, U-shaped CP, soft cleft, glossoptosis	–	Normal NIPT, karyotype, and CMA	Full-term delivery, viable
3	30	27 + 4	+	45°	PRS	Micrognathia, incomplete CP, soft cleft, glossoptosis	–	Normal NIPT, karyotype, and CMA	Full-term delivery, neonatal death
4	36	22 + 3	+	34°	PRS	Micrognathia, incomplete CP, soft cleft, glossoptosis	–	Normal NIPT, Karyotype, and CMA	Electively terminated
5	30	17 + 3	+	29°	PRS	Micrognathia, incomplete CP, soft cleft, glossoptosis	SUA	Normal NIPT	Electively terminated
6	38	25 + 2	−	43°	PRS	Micrognathia, incomplete CP, soft cleft, glossoptosis	TOF, Strephenopodia	Normal NIPT	Electively terminated
7	22	25 + 5	−	38°	PRS	Micrognathia, incomplete CP, soft cleft, glossoptosis	–	Normal NIPT	Electively terminated
8	32	18 + 1	−	26°	PRS	Micrognathia, incomplete CP, soft cleft, glossoptosis	Craniosynostosis, L-DH	Normal NIPT	Electively terminated
9	27	27 + 3	−	42°	PRS	Micrognathia, incomplete CP, soft cleft, glossoptosis	VSD, SUA	Normal NIPT	Electively terminated
10	32	24 + 1	−	42°	PRS	Micrognathia, U-shaped CP, soft cleft, glossoptosis	–	Normal NIPT, Karyotype, and CMA	Full-term delivery, viable
11	20	28 + 0	−	48°	PRS	Micrognathia, incomplete CP, soft cleft, glossoptosis	FGR	Normal NIPT	Electively terminated
12	28	23 + 2	−	46°	PRS	Micrognathia, U-shaped CP, soft cleft, glossoptosis	–	Normal NIPT, karyotype and CMA	Full-term delivery, viable
13	29	23 + 4	−	39°	PRS	Micrognathia, incomplete CP, soft cleft, glossoptosis	Tethered spinal cord, Spinal abnormality	Normal NIPT	Premature death
14	34	24 + 5	+	43°	Micrognathia, incomplete CP, soft cleft	Micrognathia, incomplete CP, soft cleft, glossoptosis	–	Normal NIPT	Full-term delivery, neonatal death

GW: gestational weeks, CMA: cytogenomic microarray analysis, NIPT: noninvasive prenatal testing, VSD: ventricular septal defect, TOF: tetralogy of Fallot, FGR: fetal growth restriction, SUA: single umbilical artery, L-DH: left diaphragmatic hernia, spinal abnormality: disordered arrangement of thoracic vertebrae, tethered spinal cord: conus medullaris located between L4-L5

### Associated abnormalities of 14 cases of PRS

The six fetuses with associated or syndromic PRS had diverse and severe malformations (Table 1). The associated malformations commonly affected the cardiac (*n* = 2), spinal (*n* = 1), and skeletal (*n* = 2) systems. After comprehensive counseling with the parents, five cases (83.3%) led to pregnancy termination. The remaining one case unfortunately ended in premature death.

In our study, five PRS cases (35.7%) were complicated by polyhydramnios as a result of swallowing difficulties secondary to the obstruction caused by severe micrognathia, while the other nine cases (64.3%) had normal amniotic fluid levels.

For every instance of PRS, the outcomes of noninvasive prenatal testing (NIPT) turned out to be normal. Karyotype analysis was conducted in six cases, showing normal karyotypes. CMA was likewise carried out in these same cases, with normal results as well. Nevertheless, in eight cases, CMA was not performed because certain families had a limited understanding of the importance of chromosomal and CMA testing (Table 1).

### Postnatal examination outcomes and respiratory management

In the group with isolated PRS, six neonates were born full-term with appropriate birth weight. Yet, all six newborns presented with varying clinical conditions and were immediately admitted to the Neonatal Intensive Care Unit (NICU). Respiratory problems that required initial or continuous intervention were observed in all six cases. Two of the newborns experienced more severe respiratory problems than expected during the prenatal period, eventually succumbing to breathing difficulties. Among the remaining four cases with favorable outcomes, three babies had a tendency to choke, while another showed frequent regurgitation but maintained normal growth. Following the doctor’s recommendations, the families of the patients decided to arrange surgeries at an appropriate time. On the contrary, in the remaining two cases of isolated PRS, the parents chose to end the pregnancies on their own.

## Discussion

PRS is considered to be a sequence that commences with micrognathia, which gives rise to glossoptosis and typically results in a broad U-shaped CP [34, 35]. CP and the posterior displacement of the tongue pose feeding problems for infants. Meanwhile, glossoptosis decreases the pharyngeal space. It also impacts the movement of the epiglottis. As a consequence, it leads to respiratory obstruction in newborns, bringing about complications such as hypoxia and breathing difficulties. These complications threaten the life of newborns [35, 36]. For newborns with PRS, regardless of whether it is isolated or not, the primary focus in treatment should be on airway obstruction and feeding difficulties. Evidently, as demonstrated previously [37], prenatal diagnosis of PRS can facilitate safe delivery and enhance perinatal care.

In our study, a total of 14 cases of PRS were identified, with an incidence rate of 0.03%. The rate is higher than that in the previous literature, which may be due to some cases suspected of having malformations in other hospitals being definitively diagnosed in our hospital during prenatal ultrasound consultations. PRS often coexists with other malformations. Specifically, 42% of the cases manifested as part of a multiple malformation syndrome. In-depth analysis of these cases showed that six cases (42.9%) were accompanied by additional malformations, predominantly cardiac, skeletal, and spinal anomalies. Among these six cases, two (33.3%) had cardiac abnormalities and two (33.3%) had skeletal malformations. Hence, during prenatal PRS diagnosis, conducting a comprehensive and detailed fetal examination is crucial. This helps rule out accompanying malformations and related syndromes, especially those affecting the cardiac and skeletal systems. It has been well established that patients with other anomalies or syndromic PRS have a higher mortality risk. A retrospective study of 181 infants demonstrated that the overall mortality rate of infants with other anomalies or syndromic PRS was 16.6%. In contrast, no deaths were reported in cases of isolated PRS [4]. In our study, the mortality rate of newborns with isolated PRS reached 33.3%, owing to serious respiratory problems during the prenatal period, which was higher than those reported in the previous literature [11, 18]. Using the four-section approach, we identified 13 cases of PRS during the second trimester. One case eluded diagnosis prenatally but was confirmed to have PRS after delivery. No false-positive results were detected. Evidently, the four-section approach for diagnosing PRS during the second trimester exhibits a sensitivity of 92.9%, specificity of 100%, positive predictive value of 100%, and negative predictive value of 91.7%.

Evidence has shown that micrognathia serves as the primary “indicator” for suspecting a PRS diagnosis. Given the efficacy of using the IFA as a diagnostic tool for PRS in MRI, we adopted IFA measurement to objectively assess micrognathia. An IFA < 50° effectively indicates micromandibular deformity, featuring a detection rate as high as 100% and a false-positive rate as low as 1.1% [23, 24]. In this study, 43 fetuses were initially suspected subjectively of having micrognathia. After undergoing reassessment with IFA measurement, 25 cases had an IFA < 50°, while 18 had an IFA > 50°. These findings were in line with the postnatal or post-mortem outcome. Thus, an IFA < 50° demonstrates remarkable predictive efficacy in identifying micrognathia, with a sensitivity and specificity both reaching 100%. Compared to previous studies, it show a lower false-positive rate in predicting micrognathia. It is widely recognized that fetuses with micromandible often develop hyperamniosis due to impaired swallowing function. In this study, five cases presented with polyhydramnios, while the remaining nine cases had normal amniotic fluid levels. Although polyhydramnios has low specificity for PRS, it is advisable to conduct a meticulous and professional ultrasound examination of the fetal facial contour and palate to rule out micrognathia and CP.

When micrognathia is suspected during the diagnostic process, the palate should be examined with great care. Prenatal detection of CP still poses a challenge that calls for significant breakthroughs. There are various types of CP, including V-shaped CP, U-shaped CP, cleft lip and palate, and submucous CP, which can present as manifestations of PRS. Notably, the U-shaped CP is a characteristic trait of classic PRS. Evidence suggests that the U-shaped CP has the strongest association with isolated PRS. Consequently, those affected usually have a relatively lower mortality risk. On the other hand, if PRS is accompanied by submucous CP, cleft lip and palate, or a seemingly normal palate, it highly indicates an underlying genetic syndrome and a significantly increased risk of death [37]. In this study cohort, postnatal inspection showed that four cases had a U-shaped CP. All these cases were categorized as isolated PRS, and their prognoses were promising.

Glossoptosis serves as a more dependable predictor of PRS compared to micrognathia. Micrognathia is a non-specific finding that can be observed in hundreds of syndromes. Even though micrognathia has long been the primary focus in the prenatal diagnosis of PRS, there has been no standardized definition for micrognathia to date. Prior research has indicated that micrognathia shows low specificity in diagnosing PRS. In contrast, glossoptosis has a sensitivity of 100%, making it more predictive of PRS than micrognathia [15]. However, technical challenges in accurately discerning the shape and position of the tongue on imaging have made the assessment of glossoptosis much more difficult. In our study, one case was prenatally diagnosed with micrognathia and incomplete CP. However, a subsequent postpartum examination verified the presence of glossoptosis. Owing to the flexion of the fetal head and neck, ultrasound imaging failed to clearly display the position of the tongue inside the oral cavity. Diagnosing glossoptosis through prenatal ultrasound still faces great difficulties. It demands dynamic and meticulous observation and is prone to misdiagnosis. When diagnosing fetuses with a small mandible, closely examining the tongue is vital. If the fetal position hinders a clear view of the tongue, it is advisable for pregnant women to do appropriate exercises to enhance the visibility of the fetal tongue. MRI has distinct advantages over ultrasound in detecting glossoptosis. MRI allows us to accurately evaluate the position of the tongue and identify potential airway obstructions. This, in turn, helps in achieving better risk stratification and offering more comprehensive consultation services to patients.

In our research, six cases had a normal karyotype and CMA findings. This outcome might be attributed to the small sample size and the absence of exon sequencing. Exon sequencing is extremely important in distinguishing PRS from other syndromes. It enables healthcare professionals to offer more precise genetic counseling to patients. Given that PRS frequently co-occurs with various syndromes, when PRS is suspected during prenatal screening, it is recommended to suggest amniocentesis for exon sequencing in future studies.

## Conclusion

Overall, the prenatal diagnosis of PRS via ultrasonography remains challenging. The four-section approach has been shown to be effective in the evaluation of PRS during second-trimester screenings. Combining glossoptosis and micrognathia assessments could significantly enhance the prenatal diagnosis accuracy of PRS. For PRS cases, comprehensive fetal and genetic exams to rule out other syndromes are pivotal. Clinicians familiar with PRS ultrasound traits can increase antenatal detection and reduce misdiagnosis, aiding clinical consultations. PRS fetuses often face neonatal breathing and feeding issues, needing multidisciplinary care. As such, antenatal PRS identification is crucial as it enables neonatologists to prepare airway interventions and intensive care, improving infant outcomes.

## Electronic supplementary material

Below is the link to the electronic supplementary material.

Supplementary Material 1

Supplementary Material 2

Supplementary Material 3

## Data Availability

Research data are confidential, and provision of patient data to other parties is not covered by ethical approval.
