# Correction: Application of four-section approach for prenatal diagnosis of Pierre robin sequence

**DOI:** 10.1007/s10396-025-01586-5

**Published:** 2025-09-30

**Authors:** Xiuling Li, Lingyan Liu, Fang Yan, Xiang Yang, Guanghui Xiu, Xudong Dong

**Affiliations:** 1 Faculty of Life Science and Technology, Kunming University of Science and Technology, Kunming, 650500 Yunnan Province People’s Republic of China; 2 Department of Obstetrics, The First People’s Hospital of Yunnan Province, The Affiliated Hospital of Kunming University of Science and Technology, 157 Jinbi Road, Xishan District, Kunming, 650032 Yunnan Province People’s Republic of China; 3 Medical School, Kunming University of Science and Technology, Kunming, 650500 Yunnan Province People’s Republic of China; 4 Department of Intensive Care Unit, The Affiliated Hospital of Yunnan University (The Second People’s Hospital of Yunnan Province), No. 176 Qingnian Road, Wuhua District, Kunming, 650021 Yunnan Province People’s Republic of China

**Correction: Journal of Medical Ultrasonics** 10.1007/s10396-025-01556-x

The article “Application of four-section approach for prenatal diagnosis of Pierre robin sequence”, written by Xiuling Li, Lingyan Liu, Fang Yan, Xiang Yang, Guanghui Xiu and Xudong Dong, was originally published under exclusive license to The Author(s), under exclusive licence to The Japan Society of Ultrasonics in Medicine 2025. As a result of the subsequent decision to publish the article under the open access model, the article’s copyright notice was changed on 16 September 2025 to © The Author(s) 2025 and the article is now distributed under a Creative Commons Attribution 4.0 International License, which permits use, sharing, adaptation, distribution and reproduction in any medium or format, as long as you give appropriate credit to the original author(s) and the source, provide a link to the Creative Commons licence, and indicate if changes were made. The images or other third party material in this article are included in the article’s Creative Commons licence, unless indicated otherwise in a credit line to the material. If material is not included in the article’s Creative Commons licence and your intended use is not permitted by statutory regulation or exceeds the permitted use, you will need to obtain permission directly from the copyright holder. To view a copy of this licence, visit http://creativecommons.org/licenses/by/4.0.

The original article has been corrected.

